# Mannitol metabolism during pathogenic fungal–host interactions under stressed conditions

**DOI:** 10.3389/fmicb.2015.01019

**Published:** 2015-09-24

**Authors:** Mukesh Meena, Vishal Prasad, Andleeb Zehra, Vijai K. Gupta, Ram S. Upadhyay

**Affiliations:** ^1^Department of Botany, Banaras Hindu UniversityVaranasi, India; ^2^Institute of Environment and Sustainable Development, Banaras Hindu UniversityVaranasi, India; ^3^Molecular Glycobiotechnology Group, Discipline of Biochemistry, School of Natural Sciences, National University of Ireland GalwayGalway, Ireland

**Keywords:** Mannitol, reactive oxygen species (ROS), mannitol dehydrogenase (MTD), mannitol-1-phosphate-5-dehydrogenase (MPD), polyols

## Abstract

Numerous plants and fungi produce mannitol, which may serve as an osmolyte or metabolic store; furthermore, mannitol also acts as a powerful quencher of reactive oxygen species (ROS). Some phytopathogenic fungi use mannitol to stifle ROS-mediated plant resistance. Mannitol is essential in pathogenesis to balance cell reinforcements produced by both plants and animals. Mannitol likewise serves as a source of reducing power, managing coenzymes, and controlling cytoplasmic pH by going about as a sink or hotspot for protons. The metabolic pathways for mannitol biosynthesis and catabolism have been characterized in filamentous fungi by direct diminishment of fructose-6-phosphate into mannitol-1-phosphate including a mannitol-1-phosphate phosphatase catalyst. In plants mannitol is integrated from mannose-6-phosphate to mannitol-1-phosphate, which then dephosphorylates to mannitol. The enzyme mannitol dehydrogenase plays a key role in host–pathogen interactions and must be co-localized with pathogen-secreted mannitol to resist the infection.

## Introduction

Mannitol, a six carbon non-cyclic sugar liquor, is a polyol commonly found in plants and fungi. In plant species, mannitol appears to be, in every way, to be the most expansive, being found in more than 70 families (Lewis and Smith, 1967; Ruijter et al., 2003). In plants, mannitol is made despite sucrose and is a phloem-translocated photoassimilate. Mannitol has various capacities in the plants including serving as a carbon stockpiling compound (Lewis, 1984), as a store of reducing power (Stacey, 1974; Loescher, 1987; Stoop and Pharr, 1992), as a compatible osmolyte (Brown and Simpson, 1972; Yancey et al., 1982) and in osmoregulation (Hellebust, 1976). Mannitol has similarly been shown to be an oxygen radical quencher both *in vitro* (Smirnoff and Cumbes, 1989) and *in vivo* (Shen et al., 1997a,b). Mannitol is the essential translocated sugar when the sucrose pool is drained (Davis and Loescher, 1990). It may moreover be incorporated in the utilization of photochemical impulses (Jennings et al., 2002).

Mannitol is the most widely recognized polyol in fungi, where it is found in spores, fruiting bodies, and mycelia (Solomon et al., 2007), and is considered to be the abundant most of all dissolvable starches inside mycelia and fruiting bodies (Lewis and Smith, 1967; Horer et al., 2001; Dulermo et al., 2009). In fungi, mannitol is a storage or a translocated carbohydrate, and is essential in spore germination under starvation conditions (Horikoshi et al., 1965; Lewis and Smith, 1967; Dijkema et al., 1985; Witteveen and Visser, 1995). Mannitol additionally extinguishes reactive oxygen species (ROS; Smirnoff and Cumbes, 1989; Chaturvedi et al., 1997; Voegele et al., 2005), prompting the speculation that it can assume a cell reinforcement part in host–pathogen interactions. As a case in point, mannitol-deficient mutants of *Cryptococcus neoformans* are less harmful than the wild type strain, probably because of the way that mannitol ensures protection against oxidative executing by phagocytic cells (Chaturvedi et al., 1996a,b).

Mannitol production and secretion are required for the pathogenicity of several fungal pathogens of both animals and plants (Chaturvedi et al., 1996a,b). In infecting tobacco, *Alternaria alternata* secretes mannitol, which is induced by host leaf extracts (Jennings et al., 1998). This observation revealed that fungal pathogens secrete mannitol to quench the ROS that mediate plant defenses. In response, pathogen-induced mannitol dehydrogenase (MTD) in the plant catabolize the pathogen’s secreted mannitol, thus protecting the plants ROS-mediated defenses. Mannitol deficient mutants of *A. alternata* created by target gene disruption had reduced pathogenicity on tobacco (Vélëz et al., 2007, 2008), confirming that mannitol production and secretion was a significant factor for pathogenicity of this fungus.

## Mannitol Biosynthesis in Fungi

**FIGURE 1:**
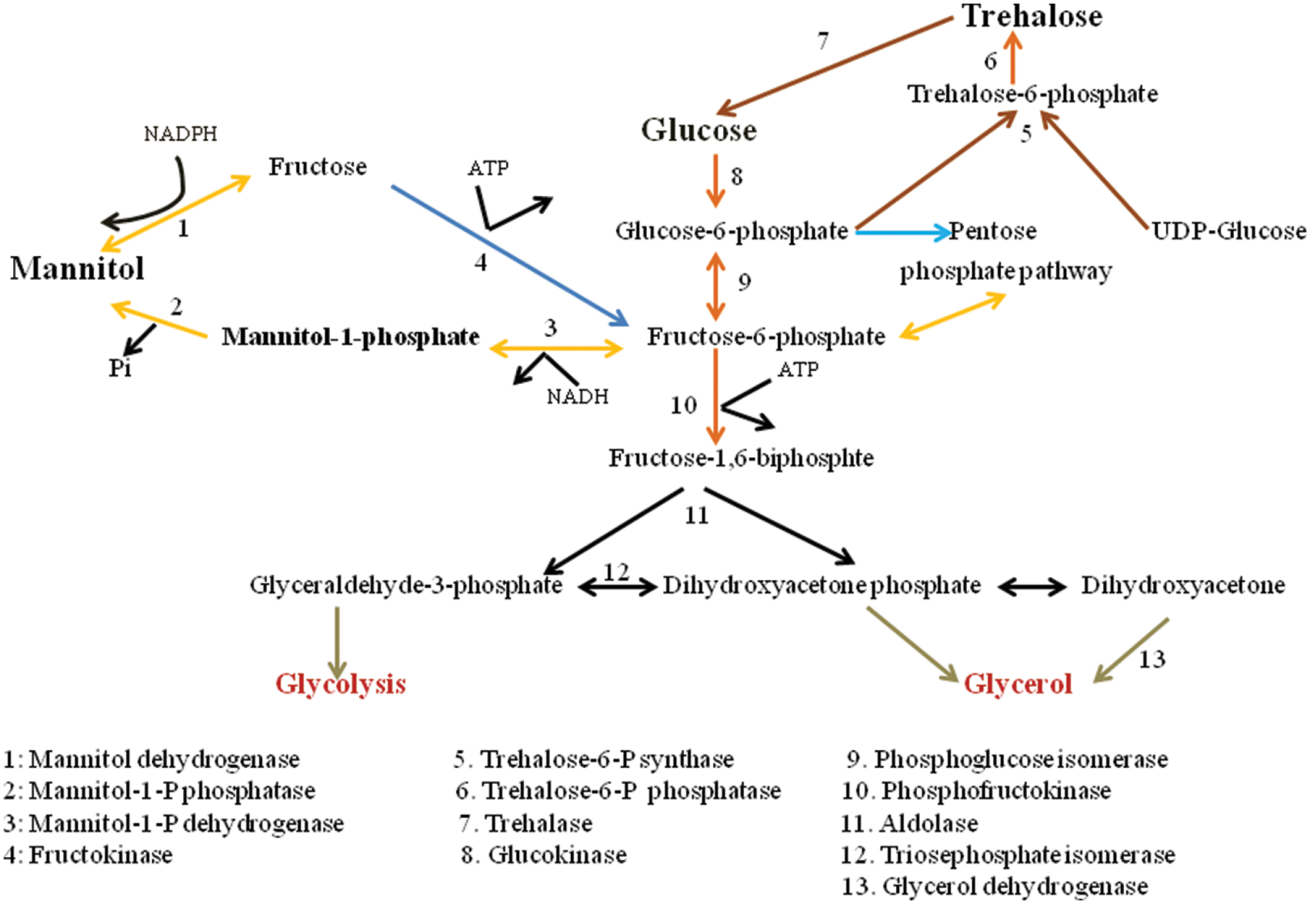
**Carbohydrate conversion and mannitol metabolic pathways in fungi**.

The metabolic pathway for mannitol biosynthesis and catabolism is well described in filamentous fungi. Mannitol metabolism in fungus is cyclical process (Hult and Gatenbeck, 1978). **Figure 1** depicts the pathways of mannitol synthesis in organisms. In this cycle, mannitol-1-phosphate 5-dehydrogenase (MPD; EC 1.1.1.17) was proposed to decrease Fructose-6-phosphate into mannitol-1-phosphate utilizing the NADH cofactor, processed by dephosphorylation by mannitol-1-phosphate phosphatase (MPP; EC 3.1.3.22), into an inorganic phosphate mannitol. Mannitol would then be oxidized to fructose by MTD (EC 1.1.1.138) utilizing the NADP^+^ cofactor. At last, fructose would be phosphorylated to fructose-6-phosphate by a hexokinase (HX; EC 2.7.1.1). Dephosphorylation of mannitol-1-phosphate into mannitol by means of MPP was portrayed as being irreversible. Thus, the proposed cycle would run in one direction with MPD as a catabolic enzyme system (Calmes et al., 2013).

The physiological functions of mannitol in fungi have been widely studied, and sometimes just as widely disputed. Mannitol serves as a store carbon source and as reservoir of reducing power (Hult et al., 1980), and other functions of mannitol have been proposed that incorporate stress resilience (Ruijter et al., 2003) and spore dispersal (Trail and Xu, 2002). The mannitol biosynthetic mutants served as a very useful model when in tobacco inoculation experiments, mutants with reduced mannitol production demonstrated an equally parallel reduction in disease symptoms (Vélëz et al., 2007).

A few fungal systems build stress resistance by collecting mannitol. The conidia of the cereal pathogen *Gibberella zeae* were promptly changed to chlamydospore-like structures (CLS) in cultures supplemented with high measures of mannitol (Reyneri, 2006; Son et al., 2012). These morphological changes in CLS, mannitol amassing may be specifically identified with expanded CLS stress resistance. Numerous parasitic species amass mannitol in their hyphae and spores, up to 10–15% of the dry weight (Witteveen and Visser, 1995). It can be hypothesized, based on information gained from model species, that this mannitol will be important for spore germination or hyphal protein during pathogenesis.

## Mannitol Biosynthesis in Plants

In higher plants more than 13 polyols are confirmed (Bieleski, 1982; Lewis, 1984), in which polyol mannitol may be the most wide spread, being found in >100 types of plants in 70 families (Lewis and Smith, 1967; Jennings, 1984; Stoop et al., 1996). **Table 1** lists plants that contain mannitol. In celery, mannitol assumes a huge part as an osmoprotectant and in addition interchange carbon and energy source (Loescher et al., 1995; Pharr et al., 1995a,b; Stoop et al., 1996; Nuccio et al., 1999; Loescher and Everard, 2000; Williamson et al., 2013). Mannitol is produced in higher plants from mannose-6-phosphate through the activity of a NADPH-mannose-6-phosphate reductase (M6PR). Mannose-6-phosphate is converted to mannitol-1-phosphate by the assistance of the M6PR enzyme, and then mannitol-1-phosphate is dephoshorylated by a phosphatase to mannitol (Rumpho et al., 1983; Loescher et al., 1992).

**Table 1:** Representative plants that contain mannitol.

Family	Species	Tissue	References
Apiaceae	*Apium graveolens* (celeric and celery) *Daucus carota* (carrot) *Pastinaca sativa* (parsnip) *Petroselinum crispum* (parsley) *Oenanthe crocata* (Hemlock Water dropwort)	Leaf, Petiole, Root Leaf, Petiole	Barker, 1955; Bourne, 1958 Pharr and Stoop, 1993 Salmon et al., 1995 Obaton, 1929; Barker, 1955 Bourne, 1958
Arecaceae	*Cocos nucifera* (coconut)	Seedlings, Embryo	Bourne, 1958
Asteraceae	*Scorzonera hispanica* (black salsify or Spanish salsify)	Root, Seeds	Patkowska and Konopinski, 2008
Bromeliaceae	*Ananas comosus* (pineapple)		Pharr and Stoop, 1993
Brassicaceae	*Brassica oleracea* (cauliflower, cabbage)	Seed	Barker, 1955; Bourne, 1958
Bromeliaceae	*Ananas sativus* (pineapple)	Fruit	Bourne, 1958
Buxaceae	*Buxus sempervirens* (common boxwood)		Zimmerman and Zeigler, 1975
Cannaceae	*Canella winterana* (Canella or Barbasco)	Stem, Bark	Pharr and Stoop, 1993
Cactaceae	*Opuntia vulgaris*		Bourne, 1958
Combretaceae	*Laguncularia racemosa* (white mangrove) *Terminalia arjuna* (Arjuna) *Terminalia chebula* (Yellow Myrobalan) *Terminalia myriocarpa* (Panisaj, Hollock) *Terminalia oliveri Brandis* (Than)	Leaves Bark Fruit	Zimmerman and Zeigler, 1975 Pharr and Stoop, 1993 Pharr and Stoop, 1993
Compositae	*Lactuca sativa* (lettuce)		Bourne, 1958
Convolvulaceae	*Ipomoea purge* (Bindweed or Jalap)		
Cucurbitaceae	*Citrullus vulgaris* (watermelon) *Cucurbita pepo* (pumpkin, squash)	Pericarp Fruit	Barker, 1955 Barker, 1955
Convolvulaceae	*Ipomea batatas* (sweet potato)	Root	
Euphorbiaceae	*Manihot utilissima* (cassava, manioc)		Zimmerman and Zeigler, 1975
Fabaceae	*Phaseolus vulgaris* (green been, French bean) *Pisum* spp. (pea) *Spartium junceum* (Spanish broom) *Cercis siliquastrum* (Judas tree, Redbud)		Barker, 1955; Bourne, 1958 Zimmerman and Zeigler, 1975 Barker, 1955; Bourne, 1958
Gnetaceae	*Ephedra distachya*		Zimmerman and Zeigler, 1975
Gramineae	*Agropyron repens* *Andropogon annulatus*		Zimmerman and Zeigler, 1975 Zimmerman and Zeigler, 1975
Liliaceae	*Asparagus officinalis* (asparagus)		Barker, 1955
Oleaceae	*Forestiera acuminate* (swamp privet) *Fraxinus americana* (white ash) *Fraxinus excelsior* (European ash) *Fraxinus ornus* (flowering ash) *Jasminum nudiflorum* (winter jasmine) *Jasminum officinale* (poet’s jessamine) *Ligustrun vulgare* (common privet) *Olea europaea* (olive) *Olea glandulifera* *Syringe vulgaris* (common lilac)	Bark, Leaf Root, Wood Leaf, Bark Leaf, Bark Bark, Flower Leaf, Bark Root, Stem	Barker, 1955; Zimmerman and Zeigler, 1975 Bourne, 1958 Barker, 1955; Zimmerman and Zeigler, 1975 Barker, 1955; Zimmerman and Zeigler, 1975 Bourne, 1958; Zimmerman and Zeigler, 1975 Barker, 1955; Loescher et al., 1992 Barker, 1955; Zimmerman and Zeigler, 1975 Trip et al., 1965
Plantanaceae	*Platanus orientalis* (oriental plane tree)	Stem, Bark, Leaves	Bourne, 1958
Rosaceae	*Prunus laurocerasus* (cherry, laurel)	Fruit	Bourne, 1958
Rubiaceae	*Coffea arabica* (coffee) *Gardenia* (several species)	Seed	Bourne, 1958 Barker, 1955; Bourne, 1958
Scrophulariaceae	*Veronica* (speedwell)	Leaf, Stem	Barker, 1955; Rumpho et al., 1983; Pedersen et al., 2007

**FIGURE 2:**
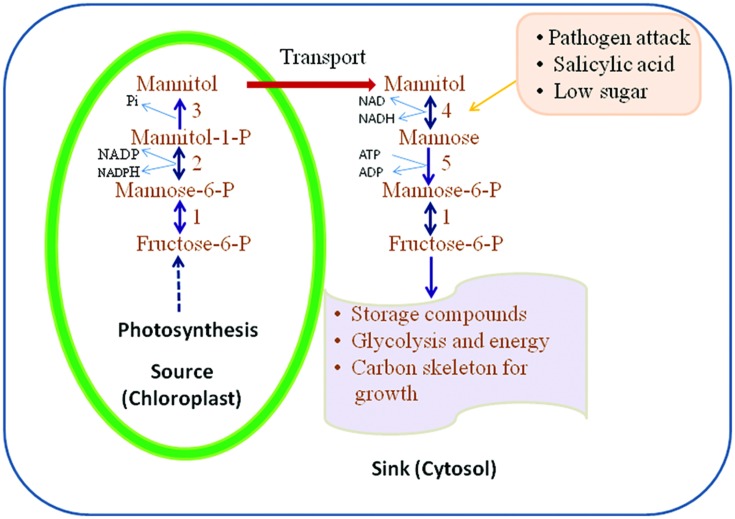
**Mannitol biosynthesis and catabolism as proposed in higher plants. 1, mannose; 6-P isomerise; 2, mannose-6-P reductase; 3, mannitol-1-phosphate dehydrogenase (M1PDA); 4, mannitol-1-dehydrogenase; 5, hexokinase (HX; adapted and modified from Stoop et al., 1996)**.

**Figure 2** illustrates the pathways of mannitol metabolism in plants. In celery, mannitol synthesis occurs in mature leaves where M6PR is localized in the cytosol of green palisade and spongy parenchyma tissues and bundle-sheath cells (Everard et al., 1993; Loescher et al., 1995). M6PR activity is regulated by light and the development stage of the plant tissue. In mature leaves of celery, the M6PR activity is high but in sink tissues such as roots and unstressed, immature leaves, no M6PR activity is detected (Everard et al., 1993, 1997; Stoop and Pharr, 1994; Jennings et al., 2002).

## Role of Mannitol and MTD during Plant–Pathogen Interactions

In fungi, mannitol plays a role in metabolism and a role in pathogenesis. In response to pathogen invasion, plants produce ROS in the extracellular space or apoplast for defense. In plants ROS are generated by a plasmalemma-embedded NADPH oxidase and/or a pH-dependent peroxidase (Bolwell and Wojtaszek, 1997). The ROS created in this “oxidative blast” serve different parts in the plant response. They serve as signals for the start of downstream resistance components, including the hypersensitive response (HR) and systemic acquired resistance (SAR). Some pathogens use strategies to circumvent these ROS-mediated defenses by the detoxification of ROS generated by host. It is reported that some plant and animal pathogenic fungi apparently use mannitol to detoxify ROS generated in the host environment.

The animal pathogen *C. neoformans* secretes large amounts of mannitol during the infection processes, and a mannitol low-producing mutant had reduced pathogenicity and oxidative stress tolerance (Chaturvedi et al., 1996a). The survival rate of *C. neoformans* was increased on addition of mannitol *in vitro* under the oxidative stress caused by ROS (Chaturvedi et al., 1996a,b). Virulent races of the tomato fungal pathogen *Cladosporium fulvum* produce and secrete mannitol during infection, while mutants unable to produce mannitol are non-pathogenic (Joosten et al., 1990). Mannitol secretion during the infection process is also reported in *Uromyces fabae* (a rust fungus), with mannitol accumulation in the apoplast paralleling high levels of fungal MTD activity (fungal mannitol biosynthesis) in the haustoria (Voegele et al., 2005). Fungal pathogen *A. alternata* also produces and secretes mannitol (Jennings et al., 1998) when treated with host plant (tobacco) extracts. Vélëz et al. (2008) reported that after treatment with the host extracts, the induction of genes for mannitol biosynthesis takes place and the pathogenicity was reduced in mannitol null mutants, which confirms that mannitol is a pathogenicity factor in fungi.

**FIGURE 3:**
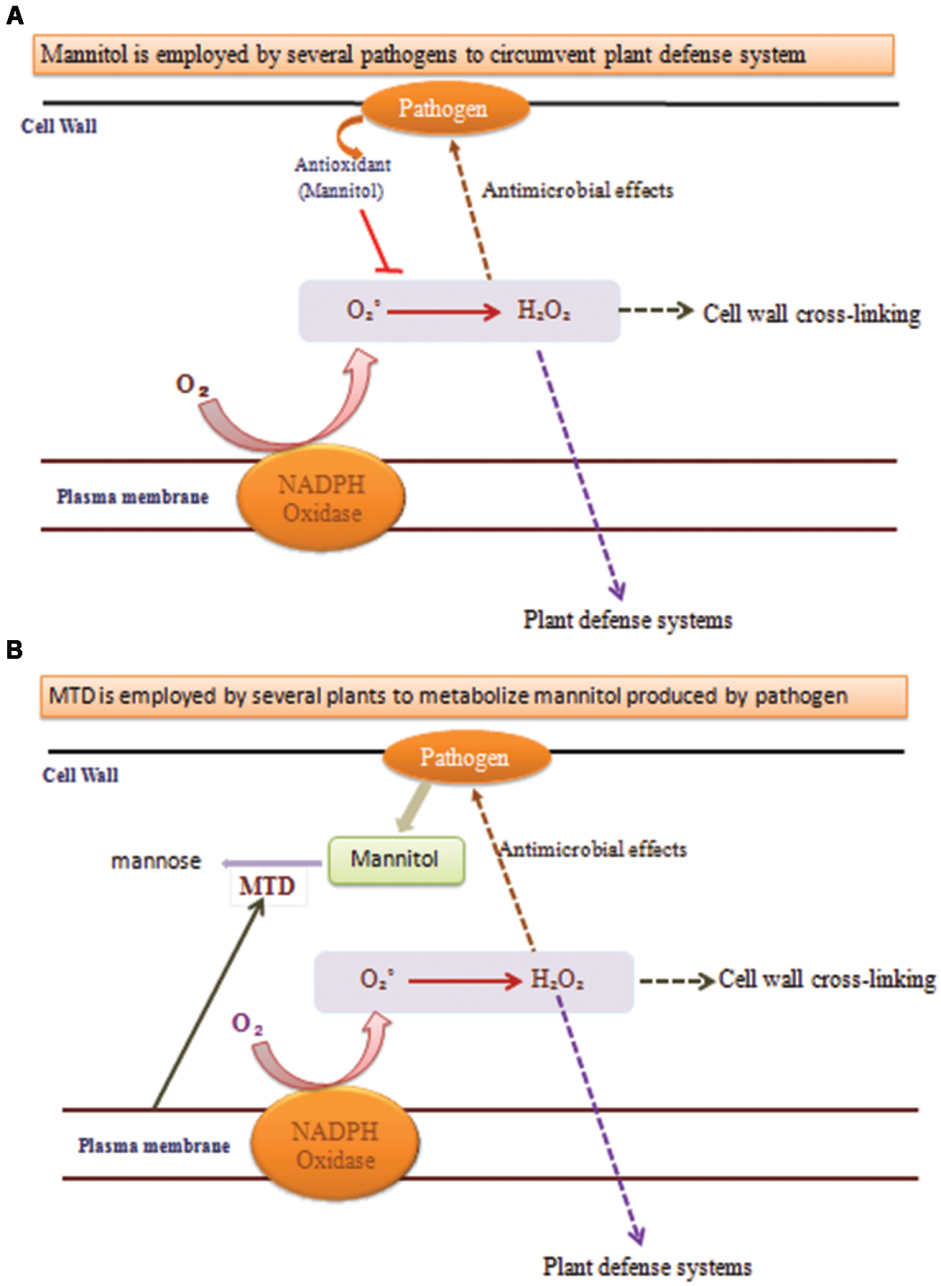
**Functions of mannitol and mannitol dehydrogenase (MTD) during plant–pathogen interactions. (A)** When pathogens uses mannitol to circumvent plant defense system it quenches the ROS production and the processes marked with dotted arrows do not occur, leading to pathogen replication. **(B)** When plants use MTD to metabolize mannitol produced by pathogens then the processes marked with dotted arrows occur and provide resistance against the pathogen.

A key indication that mannitol may have a part in plant–pathogen communications came from the observation that the celery mannitol catabolic catalyst MTD was a pathogen-induced protein in celery (**Figure 3**; Williamson et al., 1995). Tobacco does not produce mannitol, MTD and corresponding protein and RNA accumulation is induced in fungus infected tobacco (Jennings et al., 1998).

Mannitol and MTD assume noteworthy parts in photosynthesis and in salt and oxidative stress resilience. Studies likewise implicate mannitol and MTD in plant–pathogen safeguards. To discover MTD in mannitol-containing plants (e.g., celery, parsley, and snapdragon), three non-mannitol plants (tobacco, tomato, and *Arabidopsis*) have been found to contain pathogen-incited MTD, where mannitol might act as a signal molecule (Jennings et al., 1999; Chan et al., 2011; Wyatt et al., 2014). Moreover, expression of a celery MTD in tobacco confers resistance to the mannitol secreting fungus *Alternaria*. Thus, MTD appears to represent a new class of resistance gene with potential for introducing increased fungal resistance in plants (Jennings et al., 1999).

Plants use ROS both as antimicrobial operators and as signal molecules to start differing safeguard reactions. Pathogens have developed numerous modes to sidestep these barriers. Pathogens incorporate mannitol, a ROS quencher, whose creation is fundamental for pathogenicity by such differing types as the tomato pathogen *Cladosporium* and the human pathogen *Cryptococcus.* The pathogen mannitol acts in extinguishing ROS and intervening with the formation of host barriers that ought to be more impervious to assault by the plants. MTD assumes a part in host–pathogen cooperations, must be colocalized with pathogen-emitted mannitol. In early stages of plant infection, the separation of infection structures and fungal protection against extracellular ROS created by oxidative burst were related with mannitol aggregation in hyphae and conidia, separately. Amid tissue colonization, albeit the fast change of plant sugars into mannitol in response to hyphae intrusion may not be specifically connected to necrosis, the polyol likely is involved in fungal protection against intracellular isothiocyanate-induced oxidative stress (Calmes et al., 2013).

In plants, polyols (mannitol or sorbitol) play a major role in sugar transport. Polyol/monosaccharide transporters (PMTs) are involved in phloem loading and unloading (Dusotoit-Coucaud et al., 2010). Polyols being a major sugar in the metabolism of some fungi (Solomon et al., 2007), PMTs have also been recommended to assume a part in plant–fungal interactions.

## Mannitol Accumulation and Metabolism in Fungi

Fungi aggregate large amounts of polyols interacellulary, up to a several hundred millimoles every litre (Ruijter et al., 2003). *Aspergillus niger* creates various diverse polyols, including glycerol, erythriol, and D-mannitol (Witteveen and Visser, 1995). The production of the individual polyols in *A. niger* relies on the growth conditions and developmental stage. It recommends that polyols have important functions in fungal physiology. The hexitol D-mannitol is collected in numerous fungal species (Jennings, 1984). In *A. niger* conidiospores, D-mannitol is the prevalent carbon-containing compound and makes up 10–15% of the dry weight (Witteveen and Visser, 1995; Ruijter et al., 2003). The high amassings of mannitol in conidia of *A. niger* (Witteveen and Visser, 1995; Ruijter et al., 2003) and a few other organisms, for example, *Aspergillus oryzae* (Horikoshi et al., 1965), and *Aspergillus clavatus* (Corina and Munday, 1971; Ruijter et al., 2003), support a role in the survival of spores. During spore germination in *A. niger* mannitol is quickly metabolized, suggesting that it assumes a part in the capacity of carbon or reducing power (Witteveen and Visser, 1995; Ruijter et al., 2003). Correspondingly, in the basidiomycete *Agaricus bisporus* fruiting bodies, mannitol contributes up to half of the dry weight and is accepted to be a fundamental source of carbon that preserves the mushroom after harvest (Hammond and Nichols, 1975; Ruijter et al., 2003).

Mannitol is crucial for the security of spores against cell harm which happens under high temperature, drying, or increasing stress conditions. Mannitol creation is to avoid oxidative harm, for example, mannitol generation within the host by the human pathogen *C. neoformans* (Chaturvedi et al., 1996b) and secretion of mannitol by the phytopathogenic fungus *A. alternata* during plant disease (Jennings et al., 1998; Trontin et al., 2014). Interestingly, the plant increases MTD activity in light of parasitic contamination, probably for the evacuation of mannitol to balance the fungal protection mechanism (Jennings et al., 1998). In *A. niger*, mannitol is included in conidial stress resistance, especially oxidative and high-temperature stresses (Ruijter et al., 2003), and in *Stagonospora nodorum* the vicinity of mannitol is needed for asexual sporulation (Solomon et al., 2006). Since *A. niger* is a saprophyte and is pathogenic only in immuno-deficient people, it likely does not utilize mannitol generation for barrier amid development yet rather utilizes mannitol to guarantee maximal resilience of spores to survive unfavorable conditions. Numerous parasitic species aggregate trehalose and/or mannitol in their propagules, despite the fact that the levels differ (Dijksterhuis and Samson, 2002). Case in point, the trehalose level in *Aspergillus nidulans* spores is high compared to that in *A. niger* (Fillinger et al., 2001; Ruijter et al., 2003). Evidently, there is a species-particular inclination for mannitol or trehalose accumulation in conidia. In *A. nidulans* conidia, trehalose is imperative for long term spore survival and spore germination, proposing as a part of capacity carbon (Fillinger et al., 2001). The high mannitol level in *A. niger* conidia is not needed for the viability of conidiospores amid delayed stockpiling or spore germination, since inactivation of *mpdA* gene had no antagonistic impacts (Ruijter et al., 2003).

## Accumulation of Mannitol in Plants

### Abiotic Stress-imposed Mannitol Accumulation in Plants

In higher plants alditols and mannitol are osmolytes and solutes that provide resistance against various abiotic stresses (Hema et al., 2014). In a few plants mannitol and sorbitol collect in response to stress, working as osmolytes or compatible cytoplasmic solutes (Loescher, 1987). Changed tobacco plants encoding a quality mannitol-1-phosphate dehydrogenase (*mtlD*) brought about mannitol gathering, these changed tobacco plants survived, while non-changed plants were severely injured or killed when presented to 250 mol m^-3^ NaCl, indicating a function of mannitol in salt resilience (Tarczynski et al., 1993). Askari and Pepoyan (2012) reported that transgenic potato plants transformed with *mtlD* showed enhanced tolerance against salinity owing to the mannitol production due to *mtlD* activity. Similarly, a mannitol-synthesizing transgenic peanut plant has been shown to have high level of tolerance against salinity and drought stresses attributing to the abiotic stress mitigating potentials of mannitol (Bhauso et al., 2014).

A positive connection between carbon apportioning into mannitol and salt anxiety was found in celery, which creates mannitol characteristically (Kann et al., 1993). Celery grown in hydroponic nutrient solution with salinity (equal to 30% sea water) showed dry weight gain equal to control plants grown at normal nutrient level. However, fresh weight gains under high salinity were reduced as compared to control. This indicates that water content of plants decreased as salinity increased but the total assimilatory ability was unaffected. Mannitol concentration is progressively increased when total salinity of the growth solution was increased. Mannitol enhances the development of transgenic wheat submerged and saltiness stress both at the callus and entire plant level (Tilahun et al., 2003). In tobacco, these finding are similar to use the *mtlD* gene (Tarczynski et al., 1992; Karakas et al., 1997) and same as *Arabidopsis* (Thomas et al., 1995). In transgenic wheat, the measure of mannitol collection was in the low end of the concentrations reported for tobacco and *Arabidopsis.*

Numerous plant species collect polyols and cyclitols in leaves because of water stress (Noiraud et al., 2000). Mannitol and sorbitol are the most ubiquitous polyols found in plants. Mannitol is synthesised in the cytoplasm from fructose-6-phosphate under drought conditions, and these polyols accumulate up to 80% of the total solutes involved in the osmotic adjustment process of some species, like peach and celery (Lo Bianco et al., 2000). Mannitol protects thiol-regulated enzymes (e.g., Phosphoribulokinase) against hydroxyl radicals, which are abundant during the oxidative stress process associated with water stress (Shen et al., 1997a). Under osmotic stress (salt- and water-stress driven) mannitol accumulation is attributable to a reduction in the catabolism of mannitol in green tissues (Stoop and Mooibroek, 1998). Mannitol production induced by water stress has been widely observed in plant species, e.g., tomatoes (Wang et al., 2000), sugarcane (Cha-um and Kirdmanee, 2008), rice, and sorghum (Cha-um et al., 2009).

In *Olea europaea*, mannitol works as an antioxidant osmoprotectants against oxidative stress coming about because of salt/dry spell push and even sun oriented irradiance (Melgar et al., 2009; Cimato et al., 2010). Mannitol aggregation has as of late been proposed to ensure salt-treated leaves in full daylight from heat stress incited oxidative stress to a more noteworthy degree than leaves developing under incomplete shading (Cimato et al., 2010; Artur et al., 2011). Production of mannitol is useful for *C. neoformans* to oppose other environmental stresses like as heat and osmotic stresses and the mutant of *C. neoformans* performed that at minimum levels of mannitol was more vulnerable to heat and osmotic stress (Al-Fakih, 2014).

### Biotic Stress-imposed Mannitol Accumulation in Plants

The polyol mannitol extinguishes reactive oxygen species (ROS) both *in vitro* and *in vivo* (Smirnoff and Cumbes, 1989; Chaturvedi et al., 1997; Voegele et al., 2005). Studies have recommended that mannitol may be imperative in pathogenesis to cell reinforcement barriers by both plants and animals (Chaturvedi et al., 1996a,b). Jennings et al. (1998) estimated that pathogens secrete mannitol to extinguish ROS amid contamination of tobacco plants, in light of the fact that tobacco (a non-producer of mannitol) communicates a mannitol-debasing compound (MTD) when tested with parasitic elicitors and inducers of plant safeguard reactions. MTD changes over the pathogen-prompted mannitol to mannose, that is permitting the ROS intervened plant protection reaction compelling against the fungi. Transgenic tobacco plants that constitutively express MTD have increased resistance against *A. alternata* (Jennings et al., 2002). Mannitol combination happens with either sucrose blend, as in celery or with raffinose saccharide amalgamation, as in olive.

## MTD as a Pathogen-response (PR) Protein

Mannitol dehydrogenase is one catalyst in the catabolism of mannitol. Mannitol is not found in all plants, but is a photosynthetic product in over 100 species in a number of diverse families (Loescher and Everard, 2000). The regulation of its primary catabolic enzyme MTD is quite complex, responding to factors including salts and simple sugars (Williamson et al., 2002). MTD has high amino acid sequence similarity to several pathogen-response (PR) proteins of unidentified function, and its expression is regulated by the endogenous PR-proteins inducers salicylic acid (SA) and hydrogen peroxide (H_2_O_2_; Williamson et al., 1995; Zamski et al., 2001; Jennings et al., 2002). Fungal pathogens secrete mannitol to quench the ROS that mediate plant defense responses. In response, pathogen-induced MTD in the plant might then catabolize the pathogen-secreted mannitol, thus protecting the plant’s ROS-mediated defenses. MTD shows decreased activity in celery leaves exposed to high salinity, due to reduced amounts of MTD proteins (Stoop et al., 1995) and MTD transcripts (Williamson et al., 1995).

Mannitol is employed by the necrotrophic fungus *Botrytis cinerea* to overcome ROS toxicity induced during HR in plants thus making it to survive luxuriously on the necrotized dead tissue and lower plant growth and yield. Tomato plants overexpressing celery MTD owing to its PR-protein like activity exhibited enhanced resistance against *B. cinerea* (Patel et al., 2015). Also the secretion of MTD does not follow the established ER/Golgi pathway as for other PR proteins (Cheng et al., 2009; Cheng and Williamson, 2010).

## Roles of Mannitol and Other Polyols

### Osmoregulation

Polyol generation is a common feature during the growth of many organisms. In many filamentous fungi and particularly in yeasts, glycerol is the favored osmoprotectant. In the yeast *Saccharomyces cerevisiae*, glycerol contributes to the osmotic capability of the cell. Change of a *S. cerevisiae* mutant in glycerol combination with qualities for bacterial MPD and plant sorbitol-6-phosphate dehydrogenase brought about mannitol and sorbitol creation. On the other hand, strains with mutations in mannitol/sorbitol synthesis were more sensitive to salt stress than mutants changed with the gpd1 quality as demonstrated by Shen et al. (1999). They inferred that polyol collection has two capacities, encouraging osmotic alteration and supporting redox control. In *A. nidulans*, glycerol and erythritol are the major polyols included in osmoregulation. The levels of glycerol, arabinitol, and mannitol were likewise followed in *C. fulvum*-infected tomato plants under ordinary and prohibitive watering regimens for a time of eight days (Clark et al., 2003). Mannitol and malic corrosive parts in the regulation of diurnal leaf water relations were compared in “*Biancolilla*” (high-mannitol) and “*Cerasuola*” (low-mannitol) olive trees. Mannitol was the most abundant polyol distinguished at low osmotic weight, while arabinitol levels amassed at higher osmotic weight, with glycerol having a transitory accumulation (Lo Bianco and Avellone, 2014).

Mannitol is likewise one of the essential osmolytes needed for producing turgor required for ascospore release, and MTD action was seen in both asexual and sexual formative stages (Trail et al., 2002; Min et al., 2010). *G. zeae* amasses sugar alcohols (glycerol, erythritol, arabitol, and mannitol), especially against matric water stresses (Ramirez et al., 2004). Since matric potential is the significant segment of the aggregate water potential in soil and oat crop buildups, sugar alcohols, for example, mannitol may accumulate in cells cultivated under submerged conditions. Results showed that mannitol supplement in medium instigated the transformation of conidia to CLS in *G. zeae*, and a few qualities are included in this conidial adjustment. Expanded CLS imperviousness to external stresses may be gained from metabolic changes, including the accumulation of mannitol, glycogen, lipids, and chitin.

### Quencher of ROS

Mannitol and likely other sugar alcohols may be utilized to protect against ROS. Mannitol has been indicated to extinguish ROS both *in vivo* and *in vitro* (Smirnoff and Cumbes, 1989; Chaturvedi et al., 1997; Hema et al., 2014). ROS assume a significant part in pathogen resistance for both plants and animals. In animals, ROS are created by phagocytic leukocytes (macrophages/neutrophils; Rotrosen and Gallin, 1987), while in plants ROS are delivered by a NADPH oxidase confined in the plasmalamella layer (Grant and Loake, 2000). In plants, ROS have an administrative part in activating plant safeguards (e.g., lignin creation, phytoalexin production, lipid peroxidation, and the touch reaction) and additionally having antimicrobial effects (Baker and Orlandi, 1995). More confirmation that fungi use mannitol, to anticipate oxidative damage can be found in *A. alternata*, a parasitic pathogen of tobacco (*Nicotiana tabacum* L.). At the point when *A. alternata* was grown in culture medium and amended with tobacco extracts, an increment in mannitol levels and discharge was observed (Jennings et al., 1998).

Mannitol, which is made by the tomato pathogen *C. fulvum*, was found in intercellular liquids of tomato leaves infected with harmful races of *C. fulvum.* However, no mannitol was discovered if avirulent races were utilized (Joosten et al., 1990). Glucose and fructose from the plant were metabolized to mannitol by the parasite. The mannitol could then be utilized to provide energy during sporulation or could be translocated to the spores specifically (Joosten et al., 1990). Since mannitol is traded or filtered inactively into the apoplast, it could likewise have a part in ROS extinguishing.

### Storage Carbohydrate

Mannitol is implied to have a part in fungal systems as a stockpiling sugar (Lewis and Smith, 1967). Ballio et al. (1964) separated glycerol, erythritol, arabitol, mannitol, and trehalose from conidia of *Penicillium chrysogenum.* Mannitol is likewise concentrated in sclerotia of *Sclerotinia sclerotiorum, Claviceps purpurea, Claviceps nigricans*, and *Sclerotinia curreyana* (Cooke, 1969). Mannitol is found in spores of *A. oryzae, Myrothecium verrucaria, Neurospora sitophila, Neurospora crassa, A. bisporus, Sterostratum corticoides, Puccinia coronata, Puccinia graminis* f. sp. *tritici, Erysiphe graminis* f. sp. *hordei*, and *A. clavatus* (Lewis and Smith, 1967). Mannitol in the conidia of *A. oryzae* is metabolized at an early stage amid germination (Horikoshi et al., 1965). Horikoshi et al. (1965) concluded that mannitol was being utilized as the carbon hotspot for endogenous breath during the first ventures of germination, which was later managed by glucose.

In mycorrhiza, mannitol serves as sink for the translocation and storage of carbohydrate, which probably is not accessible to the host plant (Lewis and Smith, 1967; Koide et al., 2000; Calmes et al., 2013). This concept has also been applied to interaction between the fungus *C. fulvum* and tomato (*Lycopersicon esculentum* L.).

### Regulating Cofactors

Hult and Gatenbeck (1978) proposed that the mannitol cycle could be utilized to control the coenzymes NADH and NADP^+^, that is, an approach to produce NADPH to the detriment of NADH and ATP with every turn of the cycle. However, studies with the parasite *A. nidulans*, gave no support to the operation of the mannitol cycle or for NADPH generation in this fungus (Singh et al., 1988). As indicated by Singh et al. (1988) *A. nidulans* cultivated on NO_3_ as a nitrogen source would build the interest for NADPH, and accordingly create an increment in the maximal particular exercises of the enzyme in the mannitol cycle.

MTDs of the several bacterial and fungal pathogens are mannitol 2-oxidoreductases which produce fructose in the forward reaction of mannitol cycle by using either NAD^+^ (EC 1.1.1.67) or NADP^+^ (EC 1.1.1.138) as a cofactor (Voegele et al., 2005). In Basidiomycetes (Hult et al., 1980), mannitol-1-P dehydrogenase seems to be absent, so mannitol is possibly formed by direct reduction of fructose through a MTD (EC 1.1.1.67 or EC 1.1.1.138). NAD^+^-dependent MTD activity is reported in *P. graminis* f. sp. *tritici* axenic cultures (Maclean, 1990), and Clancy and Coffey (1980) have also shown NAD^+^- and NADP^+^- dependent MTD activity in *Melampsora lini* axenic cultures and uredospores (Voegele et al., 2005).

### Regulation of pH

Jennings (1984) suggested that polyols could be utilized by fungus to control their pH. Polyols could achieve this by serving as sinks or hotspots for protons, as the polyols are made (decreased) or oxidized into different starches. Ribitol accumulation occurred after mannitol consumption and during unsaturated fatty-acid degradation when a hydrogen-acceptor may be needed. At the point when the salt stress is discharged, the convergance of Cl^-^ *in vivo* diminishes (Mostaert et al., 1995), and that of fructose 6-P would be expanded by the mannitol-catabolizing pathway with MTD and HX (Karsten et al., 1997a,b; Iwamoto et al., 2003). Regulation of mannitol turn over may allow the quick reaction to a change in the natural salt situation. Since fructose 6-P is metabolically at a limb point along the mannitol pathway including glycolysis and reductive and oxidative pentose phosphate cycles, the biosynthesis of mannitol by means of M1P could likewise be controlled by the supply of F6P (Iwamoto et al., 2003). F6P could hence work as a key control component for mannitol metabolism, in addition to the regulation of M1PDH action by NaCl.

### Morphogenesis and Conidiation

Mannitol is needed for vegetative sporulation in *S. nodorum*, both *in planta* and *in vitro* (Solomon et al., 2006, 2007). Utilizing mutant strains that are not able to metabolize mannitol, the study found that subculturing of these strains on media without the polyol brought about the suspension of asexual sporulation.

The requirement of mannitol for completion of the life cycle of ascomycetes and morphogenesis in basidiomycetes is also thought to be dependent on the metabolism of mannitol, as indicated by an increase in both MTD and fructose 6-phosphate dehydrogenase activity during fruiting body development in *A. bisporus* (Stoop and Mooibroek, 1998). Mannitol is also accumulated in the unsporulated oocysts in the avian parasite *Eimeria tenella*. Mannitol enables sporulation of the oocysts outside of the host by functioning as the endogenous carbon and energy source (Allocco et al., 1999). Mannitol has been shown to be a vital factor against heat and oxidative stress in *Neosartorya fischeri* for ascospore development as well as stress resistance of conidia (Wyatt et al., 2014).

## Future Prospects

It is clear that mannitol can play an important role in plant growth and responses to diverse biotic and abiotic stresses. Possible future of mannitol for osmoprotection, efficient growth and resistance to pathogens, the engineering of plants with mannitol metabolism is a desirable goal. There is a discriminating issue, as mannitol use in sink tissues is spatially divided from its union in experienced photosynthetic tissues. There is little knowledge about the cellular and sub-cellular location of MTD or the control of MTD expression in response to environmental and metabolic factors. It is crucial to study the mannitol transport, enzyme localization and transcriptional and post-transcriptional regulation of MTD expression. Mannitol and the regulation of its production and degradation study in plants, animals and fungi is a vast topic for future research.

## Conflict of Interest Statement

The authors declare that the research was conducted in the absence of any commercial or financial relationships that could be construed as a potential conflict of interest.
